# Pretreatment Body Composition and Its Association with Pathologic Complete Response and Survival in Breast Cancer Patients Undergoing Neoadjuvant Chemotherapy: A Retrospective Cohort Study

**DOI:** 10.3390/medicina62101978

**Published:** 2026-10-13

**Authors:** Erhan Tükel, Süleyman Özkan Aksoy, Ali İbrahim Sevinç, Muhammet Berkay Sakaoğlu, Caner Bektaş, Pınar Balcı, Işıl Başara Akın, Rabia Sümeyye Arslan, Gamze Gültekin, Elif Atağ Akyürek, Yasemin Çakır, Merih Güray Durak

**Affiliations:** 1 Department of General Surgery, Faculty of Medicine, Dokuz Eylül University, 35340 İzmir, Türkiye; erhan.tkl@gmail.com (E.T.); canrbkts@gmail.com (C.B.); 2 Department of Radiology, Faculty of Medicine, Dokuz Eylül University, 35340 İzmir, Türkiye; 3 Division of Medical Oncology, Faculty of Medicine, Dokuz Eylül University, 35340 İzmir, Türkiye; 4 Department of Pathology, Faculty of Medicine, Dokuz Eylül University, 35340 İzmir, Türkiye

**Keywords:** skeletal muscle area, sarcopenic obesity, computed tomography, prognosis, treatment outcome

## Abstract

*Background and Objectives*: We investigated how pretreatment body-composition parameters relate to pathologic complete response (pCR), disease-free survival (DFS), and overall survival (OS) in women with non-metastatic breast cancer who received neoadjuvant chemotherapy (NAC). *Materials and Methods*: We retrospectively analyzed 278 women treated with NAC at a single institution between January 2010 and December 2022. On computed tomography (CT) or PET-CT, visceral adipose tissue (VAT), subcutaneous adipose tissue (SAT), skeletal muscle mass (SMM), and skeletal muscle density (SMD) were measured at the third lumbar vertebra (L3) with a semi-automated technique. Receiver operating characteristic (ROC) analysis was used to define optimal SMM cut-offs, and survival was examined by Kaplan–Meier estimation and Cox proportional-hazards regression. *Results*: The median follow-up was about 62 months. BMI was correlated with VAT (r = 0.449) and SAT (r = 0.482) but not with SMM (r = 0.075; *p* = 0.211). SMM was higher in women who attained pCR (107.4 ± 14.4 vs. 101.1 ± 16.1; *p* = 0.003). Derived by ROC analysis against distant metastasis—the event defining DFS—the SMM cut-off was 105 cm^2^ (AUC = 0.725); values below it predicted poorer DFS on both univariable (HR = 4.80; *p* < 0.001) and multivariable testing (adjusted HR = 3.83; 95% CI 1.78–8.26; *p* = 0.001). The recurrence/metastasis rate was far higher among sarcopenic obese women (*n* = 34) than non-sarcopenic obese women (*n* = 56) (41.2% vs. 10.7%; log-rank *p* = 0.0004). With adjustment for molecular subtype, greater SMM remained an independent correlate of pCR (adjusted OR 1.26 per 10-cm^2^ increment; 95% CI 1.04–1.53; *p* = 0.020). SMM was not significantly related to OS (univariable HR 1.75; *p* = 0.081). Sensitivity and specificity of the 105-cm^2^ cut-off were 92.3% and 48.2%, respectively. *Conclusions*: In this retrospective cohort, greater pretreatment skeletal muscle area was independently linked to pathologic complete response and lower area to worse disease-free survival, while no significant link emerged with overall survival. Since BMI captures muscle mass poorly, body composition obtained from CT/PET-CT may add complementary prognostic information; nevertheless, because the design was observational and the cut-offs internally derived, these hypothesis-generating results need prospective external validation before clinical adoption.

## 1. Introduction

Breast cancer is still the malignancy most often diagnosed in women and is responsible for a considerable proportion of cancer deaths [1]. Response to therapy and survival differ substantially even between patients matched for stage and molecular subtype, implying that prognosis is shaped by determinants that extend beyond standard clinicopathologic features.

Obesity has been associated with several cancers, breast cancer among them [2]. The body mass index (BMI)—introduced by Quetelet, later formalized by Keys [3,4,5], and subsequently used by the World Health Organization to categorize body weight and to declare obesity a global epidemic [6]—is still the most commonly applied obesity metric. Being an indirect ratio of weight to height, however, BMI cannot separate fat from muscle: a low value may mask a high fat fraction, whereas a high value may mask either excess fat or well-preserved muscle [7]. The “obesity paradox,” in which some individuals with elevated BMI nonetheless fare well, reflects this shortcoming [8,9].

To address these shortcomings, body-composition analysis from CT and PET-CT images has attracted growing attention in recent years [10]. Single-slice muscle and fat areas measured at L3 correspond closely to whole-body volumes and can be quantified reliably using semi-automated software [11,12]. In this way, VAT, SAT, SMM, and SMD can be extracted from images acquired for diagnosis without any extra cost.

Adipose tissue behaves as an active endocrine organ, releasing more than fifty cytokines, chemokines, and hormone-like mediators [13]. In obesity, raised insulin and insulin-like growth factor-1 lower sex hormone-binding globulin, heighten exposure to free estrogen, and thereby favor tumor growth [11,12,13]. As adipose tissue expands, local hypoxia upregulates leptin and vascular endothelial growth factor while downregulating adiponectin, shifting tumor biology toward a more aggressive phenotype [14,15]. At the same time, tumor-derived cytokines together with treatment toxicity can drive progressive muscle loss and sarcopenia in cancer patients [16]. Numerous reports have tied sarcopenia in breast cancer to higher mortality, more surgical complications, and greater chemotherapy toxicity [17,18].

Although body-composition research is expanding, evidence specific to patients undergoing NAC is still scarce. Accordingly, we set out to examine how pretreatment body composition relates to pCR, DFS, and OS among women with breast cancer who had no distant metastasis and received NAC for locally advanced disease, large tumor size, or receptor negativity.

The neoadjuvant setting is especially informative, since a standardized systemic exposure is coupled with an in vivo measure of chemosensitivity (pCR), and skeletal muscle both affects the pharmacokinetics and tolerability of chemotherapy [19,20,21] and serves as an index of host reserve. Most earlier work has used BMI or a single height-indexed muscle metric and has seldom connected the complete CT-based body-composition profile to both pathologic response and long-term survival within a single neoadjuvant cohort. We therefore hypothesized that a smaller pretreatment skeletal muscle area is associated with a lower likelihood of pCR and with poorer disease-free survival.

## 2. Materials and Methods

### 2.1. Study Design and Patient Selection

**Figure 1:**
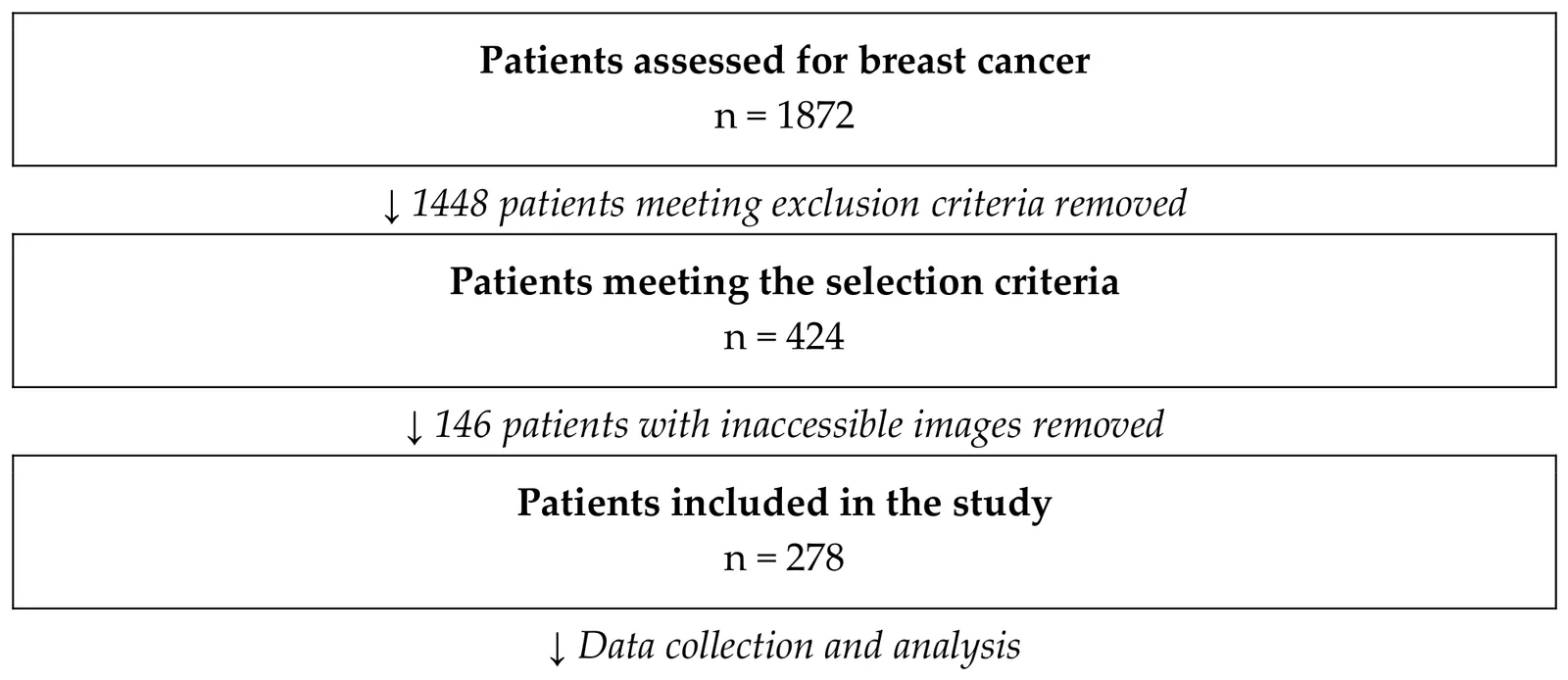
Patient selection flow diagram.

This retrospective, single-center cohort comprised women diagnosed with breast cancer at Dokuz Eylül University Faculty of Medicine Hospital between 1 January 2010 and 31 December 2022 who received NAC and then underwent surgery. Eligible participants were 18–95 years of age and had, after histopathologic diagnosis, an accessible CT or PET-CT study that included the abdomen. We excluded patients with a second malignancy and those with missing or unreliable records. No prior sample-size estimation was undertaken; rather, every eligible patient fulfilling the criteria was considered. Of 1872 patients screened, 1448 met an exclusion criterion and were removed, leaving 424; another 146 lacked accessible L3-level imaging and were likewise excluded, producing a final sample of 278 patients (Figure 1). Follow-up ended in April 2026.

### 2.2. Body Composition Measurement

Body composition was quantified for every patient from one axial slice obtained at the third lumbar vertebra (L3). These images came from the abdominal CT or 18F-FDG PET-CT studies already acquired for routine breast-cancer staging; no imaging, contrast administration, or acquisition protocol was undertaken specifically for this study. The CT and 18F-FDG PET-CT images were obtained on the routine clinical scanners used at our institution for standard staging; as this was a retrospective study spanning 2010–2022, the images originated from more than one scanner, and no imaging device was used specifically for this study. Segmentation was carried out in 3D Slicer, version 5.12.4 (open-source; https://www.slicer.org), from which the cross-sectional areas of SAT, VAT, and skeletal muscle (SMM) were obtained in cm^2^, the skeletal-muscle attenuation (SMD) in Hounsfield units (HU), and the corresponding ratios to the total cross-sectional area (SAT/TOTAL, SMM/TOTAL, VAT/SAT). In this work, SMM therefore denotes the L3 skeletal-muscle area expressed in cm^2^. Tissue boundaries were delineated using established attenuation ranges (−29 to +150 HU for skeletal muscle, −150 to −50 HU for visceral fat, and −190 to −30 HU for subcutaneous and intermuscular fat). Two readers (a professor and a radiologist) produced the measurements, which were then independently checked for accuracy by a second professor–radiologist pair; all readers were masked to outcome data, and any disagreements were settled by consensus. Single-slice L3 assessment is a well-validated and reproducible surrogate that tracks closely with whole-body muscle and adipose volumes [7,10]. Because the measurement was taken from a single slice and not scaled to height, SMM corresponds to the non-height-indexed L3 skeletal-muscle area (cm^2^).

### 2.3. Variables and Outcome Measures

The independent variables were age, BMI, menopausal status, family history, comorbidity, hormone-receptor status, tumor stage and grade, the treatments administered, and the body-composition measures. The outcome (dependent) variables were death (OS), recurrence (DFS), and distant metastasis. pCR was taken as the absence of invasive tumor and of nodal disease in the resection specimen (ypT0 ypN0); residual in situ carcinoma did not qualify as pCR. Clinical, pathologic, and imaging information was extracted from the hospital information system and the patient records, and pCR was additionally treated as a dependent variable. Here, DFS was operationalized through distant metastasis—that is, the time from surgery to the first distant metastasis; the ROC-based skeletal muscle area cut-off was therefore derived against distant metastasis, the very event on which the DFS analysis rests. OS was measured from surgery to death from any cause, with event-free patients censored at their last contact (April 2026).

### 2.4. Statistical Analysis

All statistical work was carried out in IBM SPSS Statistics, version 29.0 (IBM Corp., Armonk, NY, USA). Distributional normality was checked through skewness and kurtosis values; as most variables deviated from normality, between-group differences were examined with non-parametric methods—the Mann–Whitney U test for two-group and the Kruskal–Wallis test for multi-group comparisons. Proportions were contrasted using Pearson’s chi-square or, where appropriate, Fisher’s exact test. The sarcopenic-obesity cut-off was obtained by applying ROC analysis to the SMM distribution of obese patients. Time-to-event outcomes (DFS and OS) were summarized with Kaplan–Meier curves, compared across groups by the log-rank (Mantel–Cox) test, and modeled for independent predictors with Cox proportional-hazards regression. A two-sided *p* < 0.05 was taken as significant, and 95% confidence intervals are given where relevant. Approval for the study was obtained from the Dokuz Eylül University Non-Interventional Research Ethics Committee. Covariates reaching significance or borderline significance (*p* < 0.10) on univariable testing were carried forward into the multivariable models, together with pre-specified confounders (molecular subtype, stage, age, and menopausal status). Molecular subtype was assigned with the St. Gallen surrogate scheme (hormone-receptor status from ER/PR; HER2 positivity from immunohistochemistry 3+, or 2+ confirmed by in situ hybridization; Ki-67 split at 14%), and its relationship with pCR was tested by logistic regression. Missing values were few and addressed through complete-case analysis. The ethics committee approval number was 10921-GOA (4 May 2026).

## 3. Results

### 3.1. Patient and Tumor Characteristics

Patients had a mean age of 57.6 ± 11.5 years (range 28–88); 71.2% (*n* = 198) were over 50 years old and 73.0% (*n* = 203) were postmenopausal. The three BMI strata were evenly represented: 32.7% below 25 kg/m^2^, 34.9% between 25 and 30 kg/m^2^, and 32.4% above 30 kg/m^2^. A family history of breast cancer was recorded in 19.4%, and half of the cohort (50.4%) had at least one comorbidity. In 52.9% the tumor measured under 2 cm, and the dominant histology was no-special-type (NST) carcinoma (56.8%). Estrogen receptor was positive in 71.1% and progesterone receptor in 56.7%. Table 1 summarizes the main characteristics.

### 3.2. Relationship of BMI and Age with Body Composition

BMI showed positive correlations with VAT (r = 0.449; *p* < 0.001) and SAT (r = 0.482; *p* < 0.001) but no significant association with SMM (r = 0.075; *p* = 0.211), confirming that BMI mirrors adipose tissue while conveying nothing about muscle. Body composition shifted appreciably with age: among patients 50 years and older, VAT (129.1 ± 69.6 vs. 74.9 ± 57.3; *p* < 0.001) and the VAT/SAT ratio were higher, whereas SMM (100.9 ± 14.8 vs. 107.5 ± 17.5; *p* = 0.003) and SMD were lower. Because SAT was similar across age groups (*p* = 0.135), the age effect represented a redistribution of fat from peripheral to visceral depots rather than a change in total fat. A strong positive correlation between age and the VAT/SAT ratio reinforced this view (r = 0.545; *p* < 0.001). The VAT/SAT ratio was also higher in postmenopausal than premenopausal women (0.543 vs. 0.327; *p* < 0.001).

On stratification by BMI, the fat-related measures rose steeply with increasing BMI: VAT, SAT, total cross-sectional area (TOTAL), and the VAT/SAT ratio all differed significantly among the three groups (all *p* < 0.001). SMM, however, did not scale with BMI, increasing only slightly and non-significantly from the lowest to the highest stratum (100.6 ± 13.9 → 102.9 ± 16.5 → 104.8 ± 17.0; *p* = 0.525). The muscle-to-total-area ratio (SMM/TOTAL) actually fell significantly as BMI climbed (28.2% → 23.1% → 19.7%; *p* < 0.001). Weight gain in obese patients was thus attributable chiefly to fat, with minimal muscle accrual. These data indicate that a high BMI does not guarantee preserved muscle and that an obese patient may simultaneously be relatively muscle-depleted (sarcopenic). Table 2 presents body-composition parameters by BMI group.

### 3.3. Association with Pathologic Complete Response

pCR occurred in 26.6% of patients (*n* = 74). Women with pCR had higher SMM (107.4 ± 14.4 vs. 101.1 ± 16.1; *p* = 0.003) and a higher SMM/TOTAL ratio (*p* = 0.038), whereas VAT, SAT, and SMD did not differ between groups. Taking the survival-analysis SMM cut-off as the reference, pCR was reached in 37.2% of the SMM ≥ 105 group versus 22.3% of the SMM < 105 group (*p* = 0.008). Table 3 shows the distribution of body-composition parameters by pCR status.

After adjustment for molecular subtype in multivariable logistic regression, greater SMM stayed independently associated with pCR (adjusted OR 1.26 per 10-cm^2^ increment; 95% CI 1.04–1.53; *p* = 0.020; for SMM ≥ 105 cm^2^, adjusted OR 1.98, 95% CI 1.09–3.61). The relationship held when the tumor biomarkers were modeled separately (adjusted OR 1.31 per 10 cm^2^, 95% CI 1.07–1.60) and after additional adjustment for clinical stage, grade, and age (adjusted OR 1.31, 95% CI 1.05–1.62), showing that the skeletal-muscle-area–pCR link is independent of molecular subtype. Table 4 presents the complete model.

### 3.4. Sarcopenic Obesity and Survival

**Figure 2:**
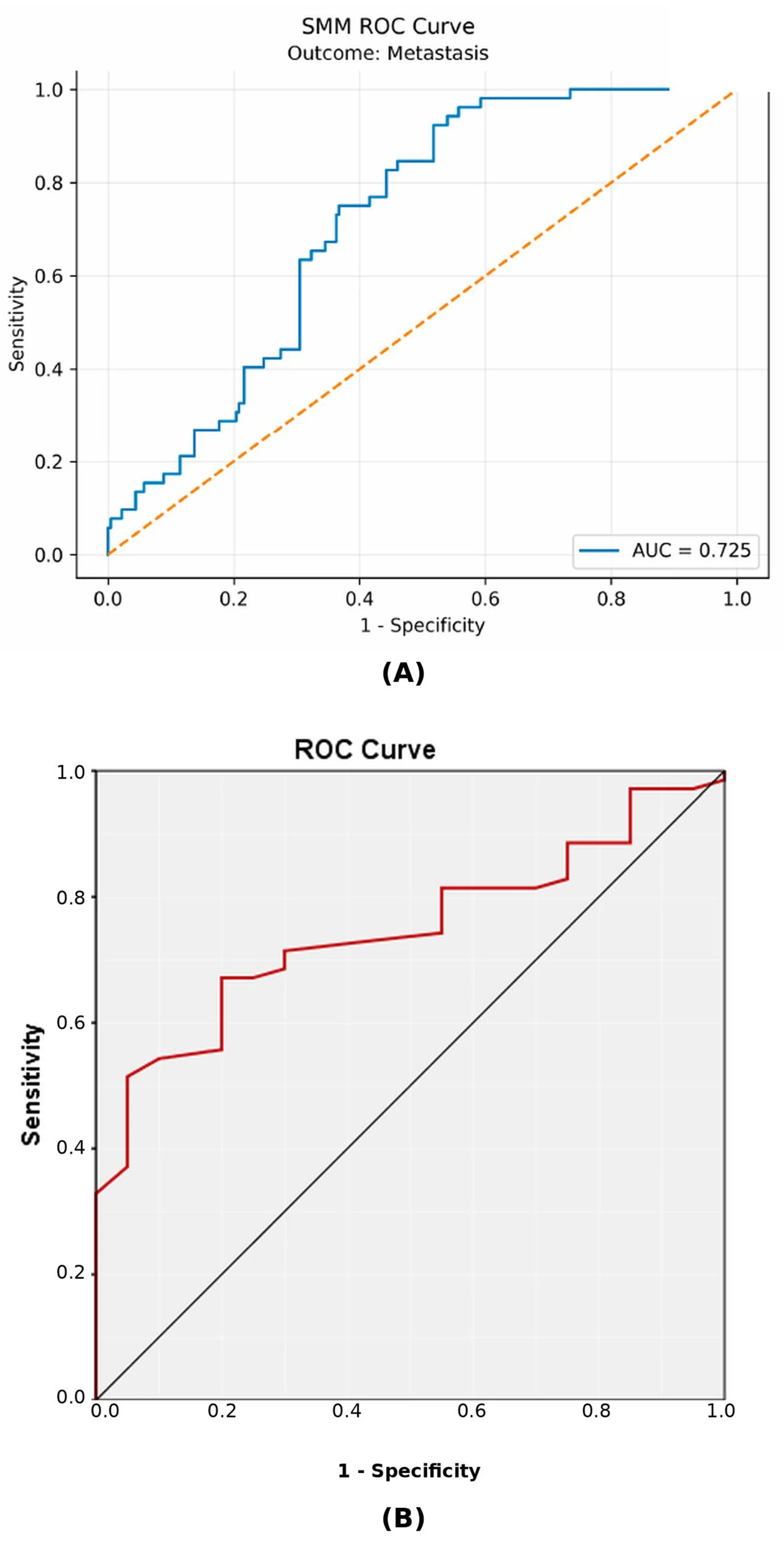
ROC curves for SMM (outcome: metastasis). (**A**) Whole cohort (AUC = 0.725; cut-off 105 cm^2^); (**B**) obese subgroup (AUC = 0.746; cut-off 95.5 cm^2^). The diagonal line represents the reference line of no discrimination (AUC = 0.5).

Sarcopenic obesity was established in two steps. First, 90 obese patients were identified using a BMI ≥ 30 kg/m^2^ criterion. Second, the SMM threshold marking sarcopenia within this obese subset was obtained by ROC analysis with metastasis as the outcome, chosen for its direct link to muscle mass. The optimal cut-off was 95.5 cm^2^, with good discrimination (AUC = 0.746; 95% CI 0.643–0.849; *p* = 0.001; sensitivity 71.4%, specificity 70%). The 34 patients (37.8%) below this value were labeled sarcopenic obese and the 56 (62.2%) above it non-sarcopenic obese. Applying the same procedure to the entire cohort yielded a cut-off of 105 cm^2^ (AUC = 0.725; sensitivity 92.3%, specificity 48.2%), which served as the reference for all patients in the subsequent survival analyses. Table 5 and Figure 2 summarizes both ROC analyses.

**Figure 3:**
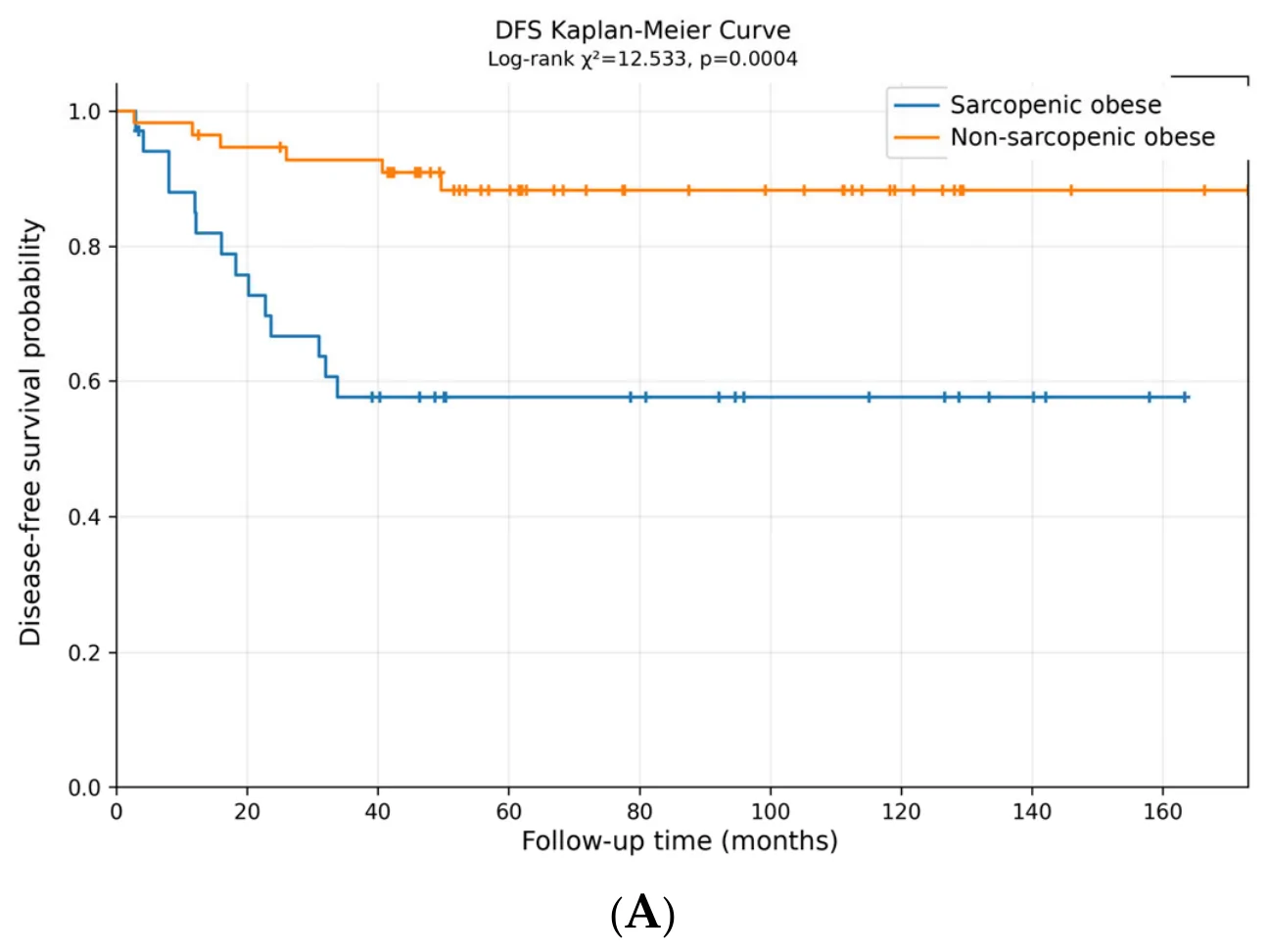

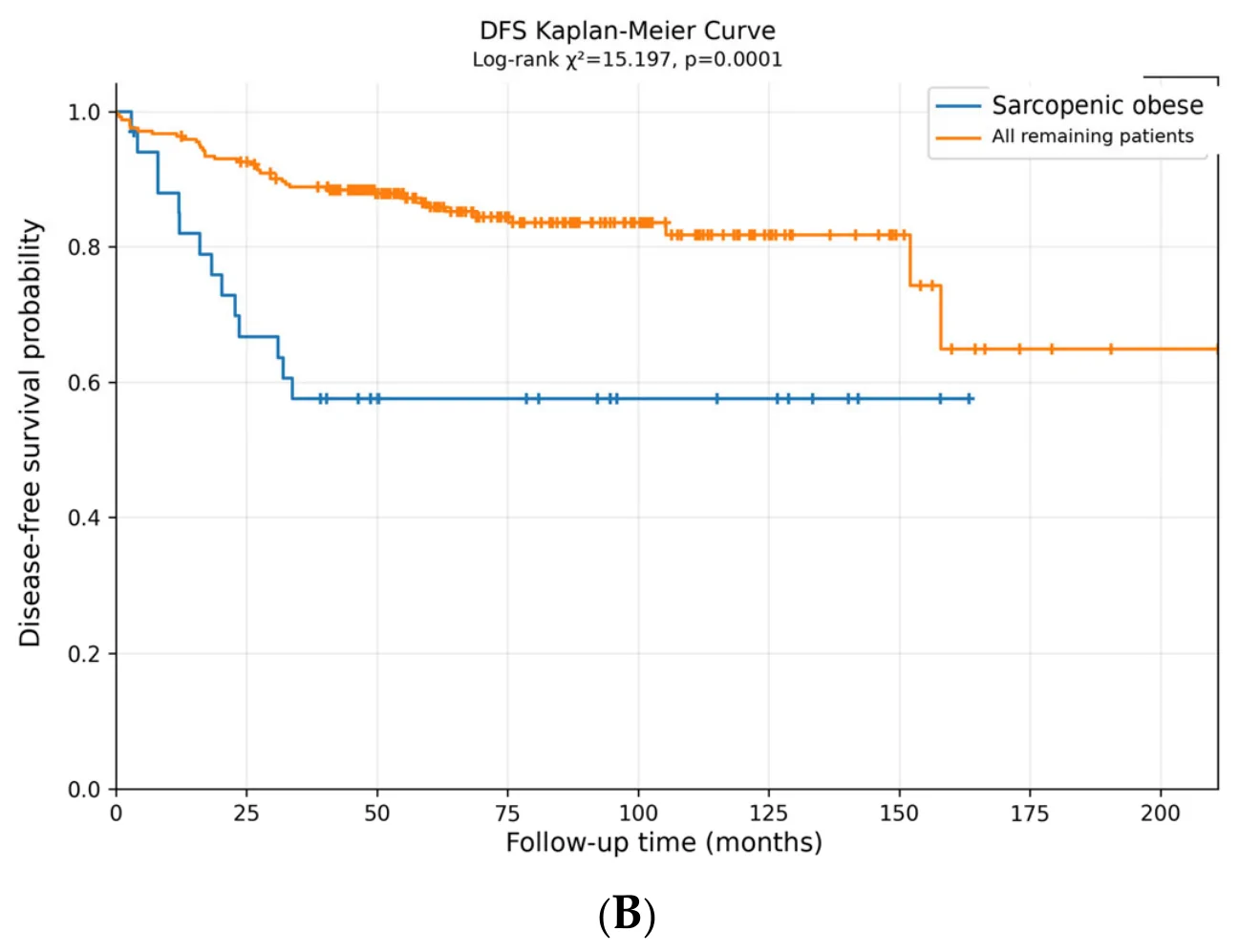
Disease-free survival (DFS) Kaplan–Meier curves. (**A**) Sarcopenic obese vs. non-sarcopenic obese (log-rank *p* = 0.0004); (**B**) sarcopenic obese vs. all remaining patients (log-rank *p* = 0.0001).

**Figure 4:**
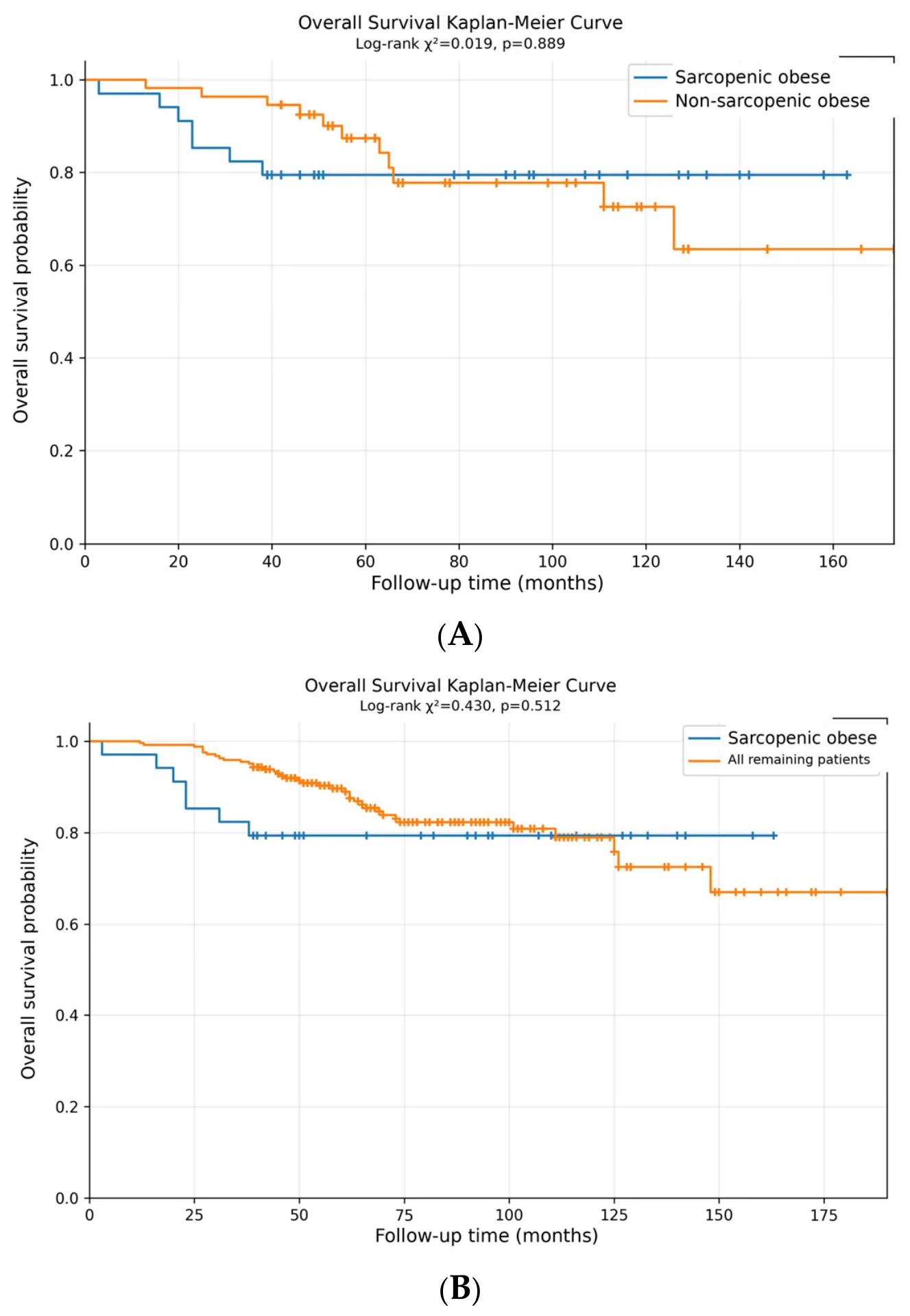
Overall survival (OS) Kaplan–Meier curves. (**A**) Sarcopenic obese vs. non-sarcopenic obese (log-rank *p* = 0.889); (**B**) sarcopenic obese vs. all remaining patients (log-rank *p* = 0.512).

Over follow-up, recurrence or metastasis arose in 41.2% (14/34) of sarcopenic obese patients compared with 10.7% (6/56) of non-sarcopenic obese patients—a significant gap (log-rank *p* = 0.0004; Figure 3A)—while OS did not differ between them (*p* = 0.889; Figure 4A). Comparing the sarcopenic obese group against all remaining patients, the DFS difference remained (log-rank *p* = 0.0001; Figure 3B), whereas the OS difference stayed non-significant (*p* = 0.512; Figure 4B). The corresponding subgroup analysis within obese patients is detailed in Table S3.

### 3.5. Prognostic Value of Skeletal Muscle Mass

Across the whole cohort, the metastasis-based SMM cut-off was 105 cm^2^ (AUC = 0.725; sensitivity 92.3%, specificity 48.2%). Consistent with this, SMM was lower in patients who developed metastasis than in those who did not (92.6 ± 11.5 vs. 105.1 ± 15.9; *p* < 0.001). On univariable Cox analysis, SMM < 105 cm^2^ was significantly tied to worse DFS (HR = 4.80; 95% CI 2.26–10.21; *p* < 0.001); tumor stage, Ki-67, lymphatic invasion, and sentinel lymph node status were likewise associated with DFS univariably. In the multivariable model, low SMM retained independent significance for DFS (adjusted HR = 3.83; 95% CI 1.78–8.26; *p* = 0.001), whereas not receiving adjuvant chemotherapy was linked to better DFS (HR = 0.45; 95% CI 0.25–0.80; *p* = 0.007; reference: patients who received adjuvant chemotherapy)—a pattern that reflects confounding by indication rather than a true protective effect, since adjuvant chemotherapy was reserved for higher-risk patients with residual disease after NAC. For OS, SMM was only borderline on univariable testing (HR = 1.75; *p* = 0.081) and non-significant in the multivariable model (adjusted HR = 1.30; 95% CI 0.68–2.49; *p* = 0.426); pathologic tumor stage alone remained an independent predictor (HR = 2.50; 95% CI 1.33–4.70; *p* = 0.004). Table 6 reports the DFS Cox results, and Table 7 the full overall-survival Cox model. Sensitivity analyses using alternative specifications of skeletal muscle area, and interaction (effect-modification) tests by obesity and menopausal status, are provided in the Supplementary Materials (Tables S1 and S2).

## 4. Discussion

In our cohort, pretreatment muscle mass was the strongest independent predictor of disease-free survival: patients with low SMM faced a several-fold higher risk of recurrence and metastasis, independently of the remaining clinicopathologic variables. This excess risk was concentrated in the sarcopenic obese subgroup, where obesity and muscle depletion coincide. For overall survival, by contrast, the contribution of muscle mass was modest, and the univariable trend lost significance once other factors were taken into account.

Perhaps the most informative aspect of our results was how little body weight alone reveals. As expected, BMI was strongly related to adipose tissue (VAT and SAT) yet entirely unrelated to muscle mass; put differently, BMI cannot tell whether a given weight is composed of muscle or of fat. Aduse-Poku et al. [22] reported the same limitation using L3-level CT and stressed the value of imaging-based assessment, and our findings reproduce that observation in a large NAC series.

Age and menopause exerted mutually reinforcing effects on body composition. As patients grow older, visceral fat accumulates while muscle wasting speeds up, and together these processes set the stage for sarcopenic obesity. Estrogen loss after menopause is recognized to drive this transition (Neilson et al. [23]). Kripa et al. [24] demonstrated that falling estradiol hampers muscle repair and promotes sarcopenia, while Trestini et al. [25] observed that visceral fat expands as subcutaneous fat contracts with age and menopause. Our data were concordant: the VAT/SAT ratio climbed markedly with age.

The greater muscle mass seen in patients who achieved pCR is biologically plausible. A sufficient muscle reserve may permit cytotoxic drugs to be given at full dose and with a potentially more favorable pharmacokinetic profile [19,20,21]. Kang et al. [26] invoked the same mechanism, linking low SMM at diagnosis to both mortality and severe neutropenia. The muscle-to-total-area ratio (SMM/TOTAL) was only weakly related to pCR, hinting that the absolute, rather than the proportional, amount of muscle governs response. In women receiving NAC for locally advanced disease, the observations of Iwase et al. accord with this interpretation [27,28].

Sarcopenic obesity describes the simultaneous presence of excess fat and diminished muscle in one patient, and the two processes act not merely additively but synergistically [29]. In our series, the sharply elevated recurrence/metastasis rate in this subgroup indicates that the driver of risk is not obesity per se but the accompanying loss of muscle. The biology is supportive: pro-inflammatory adipokines from visceral fat foster tumor proliferation and angiogenesis [13,17], while coincident muscle wasting may blunt immune responses and lower chemotherapy tolerance [19]. Gilbert and Slingerland [30] showed that obesity facilitates both local and distant spread via cytokine-mediated routes. In triple-negative disease, Gu et al. [31] identified sarcopenia as an independent determinant of DFS and OS while BMI was unrelated to survival, and the meta-analyses of Roberto et al. [32] and Zhang et al. [33] drew the same conclusion.

The central finding of this study is that low SMM was significant on univariable analysis and remained an independent predictor of DFS after multivariable adjustment. The considerably weaker hazard ratio for overall survival is itself informative: muscle loss may accompany earlier disease progression, though we did not directly examine the temporal sequence or the underlying mechanism. The lack of OS significance probably stems from the small number of events (*n* = 45). The link between not receiving adjuvant chemotherapy and better DFS must not be read as a benefit of withholding treatment; it is confounding by indication, as adjuvant chemotherapy was given preferentially to higher-risk patients with residual disease after NAC [34,35,36].

The study’s strengths include a homogeneous NAC population, a standardized CT-based measurement protocol, a comparatively large sample of 278 patients, and the simultaneous assessment of a wide panel of variables. Its main limitations are its retrospective nature, single-center scope, and few OS events. Arguably the most important limitation is that composition was captured only before treatment, so changes occurring during NAC could not be observed. On exactly this point, Jang et al. [37] demonstrated that BMI and body weight rise during therapy while muscle is lost substantially, that the weight gain conceals this loss, and that most patients sarcopenic at baseline remain so afterward—emphasizing how crucial the baseline measurement is.

The muscle threshold requires cautious interpretation on several counts. The 105-cm^2^ cut-off was obtained internally by ROC analysis within the same cohort and expressed as a non-indexed area; this may inflate its apparent prognostic strength and prevents direct comparison with height-indexed definitions of low muscle mass, such as a skeletal muscle index < 38.5 cm^2^/m^2^ in women [19] or the BMI-stratified thresholds of Martin et al. [38]. Recent breast-cancer studies that apply such thresholds describe inconsistent associations with pCR, from an independent predictive role [39] to none, and a similar difficulty in exporting predefined cut-offs is reported in other malignancies such as retroperitoneal sarcoma [40]. Beyond muscle quantity, skeletal muscle density captures muscle quality and has lately been associated with pCR in triple-negative breast cancer [41]; we interpret our SMD results in that light.

Further limitations deserve mention. As muscle strength and physical performance were not measured, our classification denotes CT-defined low muscle mass rather than consensus-defined sarcopenia, and we use the term accordingly. Excluding 146 patients without measurable L3 images may introduce selection bias; retrospective data extraction carries risks of information bias and missing data; residual confounding (molecular subtype, treatment, stage, age, menopausal status) cannot be ruled out; imaging accrued over many years may be heterogeneous; and the adjuvant-chemotherapy analysis is subject to confounding by indication and to temporal bias.

## 5. Conclusions

In this retrospective, single-center cohort of breast cancer patients given neoadjuvant chemotherapy, pretreatment body composition was related to treatment response and disease-free survival but not to overall survival. A larger L3 skeletal muscle area was independently associated with a higher chance of pathologic complete response, and a smaller area with poorer disease-free survival—including within the sarcopenic obese subgroup—while no significant association emerged for overall survival.

These observations suggest that body composition obtained from CT or 18F-FDG PET-CT—images already acquired during routine work-up and therefore at no extra cost—may add prognostic information beyond body mass index in this setting. However, given the retrospective, single-center design and the internally derived, non-height-indexed cut-offs, the associations should be viewed as hypothesis-generating rather than as a basis for clinical decisions. Rather than supporting immediate clinical use, these findings may help inform and open the way for future prospective, multicenter, externally validated studies required to confirm them and to explore whether nutritional or exercise interventions can modify these outcomes.

## Figures and Tables

**Table 1:** Baseline Clinical and Pathologic Characteristics of the Patients (*N* = 278).

Variable	Category	*n* (%)
Age	≤50	80 (28.8)
	>50	198 (71.2)
Menopausal status	Premenopausal	75 (27.0)
	Postmenopausal	203 (73.0)
BMI	≤25 kg/m^2^	91 (32.7)
	25.1–29.9 kg/m^2^	97 (34.9)
	≥30 kg/m^2^	90 (32.4)
Marital status *	Single	13 (4.7)
	Married	207 (75.5)
	Widowed	20 (7.3)
	Divorced	34 (12.4)
Family history of breast cancer	Yes	54 (19.4)
	No	224 (80.6)
Comorbidity	Yes	140 (50.4)
	No	138 (49.6)
Tumor size	<2 cm	147 (52.9)
	2–5 cm	103 (37.1)
	>5 cm	28 (10.1)
Pathologic diagnosis	NST	158 (56.8)
	Special type	75 (27.0)
	Mixed	45 (16.2)
Pathologic grade †	Grade I	24 (8.8)
	Grade II	162 (59.3)
	Grade III	87 (31.9)
Pathologic tumor stage	Tx	5 (1.8)
	ypT0	80 (28.8)
	ypT1	127 (45.7)
	ypT2	50 (18.0)
	ypT3	9 (3.2)
	ypT4	7 (2.5)
Pathologic nodal stage ‡	ypN0	124 (45.1)
	ypN1	82 (29.8)
	ypN2	49 (17.8)
	ypN3	20 (7.3)
Estrogen receptor	Positive	197 (71.1)
	Negative	80 (28.9)
Progesterone receptor	Positive	154 (56.7)
	Negative	123 (43.3)
Ki-67 §	≤14%	61 (22.7)
	>14%	208 (77.3)
Lymphatic invasion	Present	108 (38.8)
	Absent	170 (61.2)
Vascular invasion	Present	50 (18.0)
	Absent	228 (82.0)
Neural invasion	Present	29 (10.4)
	Absent	249 (89.6)
Pathologic complete response (pCR)	Yes	74 (26.6)
	No	204 (73.4)

BMI: body mass index; NST: no special type; pCR: pathologic complete response. Percentages are based on valid data: * marital status *n* = 274; † pathologic grade *n* = 270; ‡ pathologic nodal stage *n* = 275; § Ki-67 *n* = 269.

**Table 2:** Comparison of Body Composition Parameters by BMI Group.

Parameter	BMI ≤ 25 (*n* = 91)	BMI 25.1–29.9 (*n* = 97)	BMI ≥ 30 (*n* = 90)	*p*
VAT	76.7 ± 54.9	116.8 ± 68.2	147.1 ± 70.1	**<0.001**
SAT	190.5 ± 70.9	234.1 ± 73.4	289.2 ± 99.0	**<0.001**
SMM	100.6 ± 13.9	102.9 ± 16.5	104.8 ± 17.0	0.525
SMD	25.9 ± 11.3	25.6 ± 13.6	22.1 ± 9.0	0.054
VAT/SAT	39.3 ± 23.4	52.2 ± 33.5	53.6 ± 26.0	**<0.001**
TOTAL	408.1 ± 204.0	468.5 ± 123.8	557.2 ± 153.7	**<0.001**
SAT/TOTAL (%)	48.7 ± 10.2	49.4 ± 9.5	51.0 ± 9.6	0.177
SMM/TOTAL (%)	28.2 ± 9.9	23.1 ± 6.7	19.7 ± 5.7	**<0.001**

Data are presented as mean ± standard deviation; between-group comparisons used the Kruskal–Wallis test. BMI is expressed in kg/m^2^. VAT: visceral adipose tissue; SAT: subcutaneous adipose tissue; SMM: skeletal muscle mass; SMD: skeletal muscle density; TOTAL: total cross-sectional area.

**Table 3:** Body Composition Parameters by Pathologic Complete Response Status.

Parameter	pCR Present (*n* = 74)	pCR Absent (*n* = 204)	*p*
VAT	106.9 ± 65.6	115.9 ± 72.3	0.404
SAT	237.7 ± 80.2	237.6 ± 94.6	0.738
SMM	107.4 ± 14.4	101.1 ± 16.1	**0.003**
SMD	25.8 ± 14.4	24.2 ± 10.5	0.702
SMM/TOTAL (%)	24.9 ± 7.0	23.2 ± 8.8	**0.038**

**Table 4:** Multivariable logistic regression for pathologic complete response (*n* = 271; 71 events; adjusted for molecular subtype). OR, odds ratio; ref, reference category.

Variable	Adjusted OR (95% CI)	*p*
**SMM (per +10 cm^2^)**	**1.26 (1.04–1.53)**	**0.020**
Molecular subtype (ref: Luminal A)		
Luminal B	6.13 (1.35–27.95)	0.019
HER2-positive	19.08 (4.33–84.09)	<0.001
Triple-negative	17.01 (3.54–81.64)	<0.001

Data are shown as mean ± standard deviation. VAT: visceral adipose tissue; SAT: subcutaneous adipose tissue; SMM: skeletal muscle mass; SMD: skeletal muscle density.

**Table 5:** ROC Analysis Results for Determining SMM Cut-off Values.

Group (Outcome: Metastasis)	AUC	Cut-Off (SMM)	Sensitivity/Specificity	*p*
Obese patients (BMI ≥ 30; *n* = 90)	0.746	95.5	71.4%/70%	**0.001**
Whole cohort (*n* = 278)	0.725	105	92.3%/48.2%	—

The 95% CI for the obese subgroup was 0.643–0.849. Within each group, an SMM value below the cut-off defined relative muscle depletion (sarcopenia). AUC: area under the curve; CI: confidence interval; SMM: skeletal muscle mass.

**Table 6:** Cox Regression Analysis for Disease-Free Survival.

Variable	Univariable HR (95% CI)	*p*	Multivariable HR (95% CI)	*p*
Pathologic tumor stage > T1	2.15 (1.23–3.77)	0.007	1.70 (0.92–3.15)	0.090
Ki-67 > 14%	3.25 (1.17–9.03)	0.024	2.66 (0.92–7.68)	0.072
Lymphatic invasion	2.01 (1.15–3.50)	0.014	1.21 (0.65–2.26)	0.541
Sentinel lymph node negative	0.42 (0.19–0.93)	0.033	0.51 (0.22–1.19)	0.119
No adjuvant chemotherapy	0.48 (0.27–0.82)	0.008	**0.46** **(0.26–0.81)**	**0.007**
SMM < 105 cm^2^	4.80 (2.26–10.21)	<0.001	**3.83 (1.78–8.26)**	**0.001**

HR: hazard ratio; CI: confidence interval; SMM: skeletal muscle mass. The disease-free-survival event was distant metastasis. The reference category for adjuvant chemotherapy was patients who received it. In the multivariable model, only SMM < 105 cm^2^ and adjuvant-chemotherapy status were independent predictors of DFS.

**Table 7:** Cox regression analysis for overall survival (*n* = 269; 45 events; nine patients with missing Ki-67 status were excluded from the 278-patient cohort). Reference for adjuvant chemotherapy: patients who received adjuvant chemotherapy. Pathologic tumor stage was the only independent predictor of OS.

Variable	Univariable HR (95% CI)	*p*	Multivariable HR (95% CI)	*p*
SMM < 105 cm^2^	1.75 (0.93–3.30)	0.081	1.30 (0.68–2.49)	0.426
Pathologic tumor stage > T1	2.54 (1.41–4.59)	0.002	**2.50 (1.33–4.70)**	**0.004**
Ki-67 > 14%	2.35 (0.84–6.58)	0.105	2.18 (0.75–6.37)	0.152
Lymphatic invasion	1.78 (0.98–3.23)	0.059	1.29 (0.68–2.43)	0.432
No adjuvant chemotherapy	0.63 (0.34–1.15)	0.130	0.70 (0.38–1.29)	0.254

## Data Availability

The data presented in this study are available upon request from the corresponding author. The data are not publicly available due to privacy restrictions.

## Supplementary Materials

The following supporting information can be downloaded at: https://www.mdpi.com/article/10.3390/medicina62101978/s1, Table S1: sensitivity analyses of skeletal muscle area under different modeling specifications; Table S2: interaction (effect-modification) tests; Table S3: subgroup analysis in obese patients (sarcopenic obesity).

## Abbreviations

The following abbreviations are used in this manuscript:

NAC	neoadjuvant chemotherapy
pCR	pathologic complete response
DFS	disease-free survival
OS	overall survival
BMI	body mass index
CT	computed tomography
PET-CT	positron emission tomography–computed tomography
L3	third lumbar vertebra
VAT	visceral adipose tissue
SAT	subcutaneous adipose tissue
SMM	skeletal muscle mass
SMD	skeletal muscle density
TOTAL	total cross-sectional area
ROC	receiver operating characteristic
AUC	area under the curve
HR	hazard ratio
CI	confidence interval
NST	no special type
