# Supplementary material for: Pretreatment Body Composition and Its Association with Pathologic Complete Response and Survival in Breast Cancer Patients Undergoing Neoadjuvant Chemotherapy: A Retrospective Cohort Study
Source: Medicina (Kaunas). 2026 Oct 13;62(10):1978. doi: 10.3390/medicina62101978 (PMC13706991; doi:10.3390/medicina62101978)
Supplement: Supplementary file 1 [file medicina-62-01978-s001.zip › medicina-4547181-supplementary.pdf]

Supplementary Materials

"Pretreatment Body Composition Status and Its Association with Pathologic Complete Response and Survival in Breast Cancer Patients Undergoing Neoadjuvant Chemotherapy: A Retrospective Cohort Study"

These supplementary analyses were performed to address the reviewers' requests regarding the robustness (sensitivity analyses) and potential effect modification (interaction tests) of the associations between skeletal muscle area and study outcomes. All analyses used the same dataset as the main manuscript. Disease-free survival (DFS) was defined by distant metastasis, consistent with the main analysis.

S1. Sensitivity analyses: alternative specifications of skeletal muscle area

To confirm that the associations were not dependent on the internally derived 105-cm² cut-off, skeletal muscle area (SMM) was modelled in three ways for each outcome: as a continuous variable (per +10 cm² and per +1 standard deviation) and as a binary variable (< 105 cm² vs ≥ 105 cm²). The direction and significance of the associations were consistent across all specifications.

Table S1. Association of skeletal muscle area with each outcome under different modelling specifications.

Outcome	SMM per +10 cm²	SMM per +1 SD	SMM < 105 cm² (vs ≥ 105)
pCR — OR (95% CI)	1.29 (1.08–1.53); p=0.004	1.49 (1.14–1.96); p=0.004	0.45 (0.26–0.77); p=0.003
DFS — HR (95% CI)	0.62 (0.51–0.74); p<0.001	0.46 (0.34–0.62); p<0.001	4.80 (2.26–10.21); p<0.001
OS — HR (95% CI)	0.81 (0.67–0.98); p=0.027	0.71 (0.53–0.96); p=0.027	1.75 (0.93–3.30); p=0.081

pCR, pathologic complete response; DFS, disease-free survival (event: distant metastasis); OS, overall survival; OR, odds ratio (logistic regression); HR, hazard ratio (Cox regression). For the continuous variables a value > 1 (OR) or < 1 (HR) indicates that higher muscle area is associated with a higher probability of pCR and a lower risk of a DFS/OS event, respectively. For the dichotomous variable, estimates are for SMM < 105 cm² (reference ≥ 105 cm²). The association of low muscle mass with pCR and DFS was significant and directionally consistent across all specifications; for OS, the continuous measure was nominally significant whereas the dichotomous measure was not.

S2. Interaction (effect-modification) tests

Potential effect modification of the skeletal muscle area associations by obesity (body mass index ≥ 30 kg/m²) and by menopausal status was assessed by adding a multiplicative interaction term to the corresponding regression model and comparing nested models with a likelihood-ratio (LR) test. No interaction reached statistical significance, indicating that the association of skeletal muscle area with the outcomes did not differ significantly across these subgroups.

Table S2. Likelihood-ratio tests for interaction between skeletal muscle area (per +10 cm²) and obesity or menopausal status.

Interaction term	Outcome	LR χ^2 (df = 1)	p
SMM × obesity (BMI ≥ 30)	pCR	2.44	0.119
SMM × menopausal status	pCR	1.30	0.255
SMM × obesity (BMI ≥ 30)	DFS	0.01	0.939

SMM × menopausal status	DFS	2.55	0.110
SMM × obesity (BMI ≥ 30)	OS	0.00	0.992

LR, likelihood-ratio. None of the interaction terms was statistically significant (all $p > 0.10$), indicating no significant effect modification by obesity or menopausal status.

S3. Subgroup analysis within obese patients (sarcopenic obesity)

As a subgroup/sensitivity analysis, patients with obesity (BMI ≥ 30 kg/m^2, n = 90) were analysed separately using the ROC-derived sarcopenic-obesity threshold (SMM < 95.5 cm^2; see main-text **Table 5**). Within this subgroup, sarcopenic obesity was strongly associated with worse disease-free survival.

Table S3. Disease-free survival in obese patients according to sarcopenic obesity status.

Subgroup (n = 90 obese)	DFS — HR (95% CI)	p
Sarcopenic obese (SMM < 95.5 cm^2) vs non-sarcopenic obese	4.80 (1.84–12.52)	0.001

Consistent with the log-rank comparison reported in the main manuscript ($p = 0.0004$).

All analyses were performed in Python (Cox proportional-hazards and logistic regression with likelihood-ratio tests); results were cross-checked against the SPSS 29.0 outputs of the primary study.
